# Development and Validation of a Clinical-Based Signature to Predict the 90-Day Functional Outcome for Spontaneous Intracerebral Hemorrhage

**DOI:** 10.3389/fnagi.2022.904085

**Published:** 2022-05-09

**Authors:** Xiaoyu Huang, Dan Wang, Qiaoying Zhang, Yaqiong Ma, Shenglin Li, Hui Zhao, Juan Deng, Jingjing Yang, JiaLiang Ren, Min Xu, Huaze Xi, Fukai Li, Hongyu Zhang, Yijing Xie, Long Yuan, Yucheng Hai, Mengying Yue, Qing Zhou, Junlin Zhou

**Affiliations:** ^1^Department of Radiology, Lanzhou University Second Hospital, Lanzhou, China; ^2^Second Clinical School, Lanzhou University, Lanzhou, China; ^3^Key Laboratory of Medical Imaging of Gansu Province, Lanzhou, China; ^4^Gansu International Scientific and Technological Cooperation Base of Medical Imaging Artificial Intelligence, Lanzhou, China; ^5^Department of Radiology, Xi’an Central Hospital, Xi’an, China; ^6^Department of Radiology, Gansu Provincial Hospital, Lanzhou, China; ^7^GE Healthcare, Beijing, China

**Keywords:** stroke, spontaneous intracerebral hemorrhage, Glasgow Coma Scale, outcome, perihematomal edema

## Abstract

We aimed to develop and validate an objective and easy-to-use model for identifying patients with spontaneous intracerebral hemorrhage (ICH) who have a poor 90-day prognosis. This three-center retrospective study included a large cohort of 1,122 patients with ICH who presented within 6 h of symptom onset [training cohort, *n* = 835; internal validation cohort, *n* = 201; external validation cohort (center 2 and 3), *n* = 86]. We collected the patients’ baseline clinical, radiological, and laboratory data as well as the 90-day functional outcomes. Independent risk factors for prognosis were identified through univariate analysis and multivariate logistic regression analysis. A nomogram was developed to visualize the model results while a calibration curve was used to verify whether the predictive performance was satisfactorily consistent with the ideal curve. Finally, we used decision curves to assess the clinical utility of the model. At 90 days, 714 (63.6%) patients had a poor prognosis. Factors associated with prognosis included age, midline shift, intraventricular hemorrhage (IVH), subarachnoid hemorrhage (SAH), hypodensities, ICH volume, perihematomal edema (PHE) volume, temperature, systolic blood pressure, Glasgow Coma Scale (GCS) score, white blood cell (WBC), neutrophil, and neutrophil-lymphocyte ratio (NLR) (*p* < 0.05). Moreover, age, ICH volume, and GCS were identified as independent risk factors for prognosis. For identifying patients with poor prognosis, the model showed an area under the receiver operating characteristic curve of 0.874, 0.822, and 0.868 in the training cohort, internal validation, and external validation cohorts, respectively. The calibration curve revealed that the nomogram showed satisfactory calibration in the training and validation cohorts. Decision curve analysis showed the clinical utility of the nomogram. Taken together, the nomogram developed in this study could facilitate the individualized outcome prediction in patients with ICH.

## Introduction

There is a large difference between the incidence and morbidity rates of spontaneous intracerebral hemorrhage (ICH), with ICH accounting for only 20% of all stroke types but having a 1-month mortality rate of almost 40% (Feigin et al., 2009; van Asch et al., 2010). There remains no effective treatment for ICH. Hematoma expansion (HE) is an independent risk factor for ICH (Dowlatshahi et al., 2011; Demchuk et al., 2012), however, interventions for reducing HE do not provide a definitive therapy with a significant impact on the functional outcome (Anderson et al., 2013; Qureshi et al., 2016; Sprigg et al., 2018). Despite the bottlenecks in ICH treatment, there has been extensive research on ICH (including prognosis prediction) to yield prompt treatment suggestions for patients according to their possible prognosis outcomes. Non-contrast computed tomography (NCCT) is the first-choice imaging examination for patients with ICH (Latchaw et al., 2009) and allows rapid diagnosis, rapid treatment guidance, and improved treatment outcomes. Therefore, there has been extensive research on outcome prediction and HE in patients based on CT makers (Wada et al., 2007; Orito et al., 2016; Li et al., 2017; Deng et al., 2020; Peeters et al., 2021). CT-based imaging makers have shown good prediction accuracy for prognosis or HE prediction in patients with ICH. However, there remains no consensus regarding the methods and diagnostic criteria for identifying these markers; further, there are no externally validated prediction models. Additionally, the evaluation process of these markers is largely subjective; therefore, their predictive accuracy in real-world clinical practice is relatively reduced. A review (Morotti et al., 2019) was conducted regarding the detection, interpretation, reporting, and status of these markers, which may extensively improve their clinical application. With the advent of artificial intelligence (AI), radiomic methods have been extensively applied to predict the prognosis and HE of patients with ICH (Shen et al., 2018; Xie et al., 2020; Xu et al., 2020; Pszczolkowski et al., 2021; Song et al., 2021). However, this method remains in the research phase; moreover, its predictive performance should be further verified. Upon admission of patients with ICH, laboratory test results and clinical characteristics are obtained, which are objective and timely.

We hypothesized that clinical information and laboratory data may facilitate the prediction of 90-day functional outcomes in patients with ICH. Accordingly, we aimed to develop and independently validate a nomogram for detecting poor 90-day prognosis in patients with ICH.

## Materials and Methods

### Patients With Intracerebral Hemorrhage

This retrospective study was conducted in accordance with the institutional review boards (IRBs) of Lanzhou University Second Hospital, which also approved this study protocol. This study patient consent was not required in this observational study registry.

**FIGURE 1:**
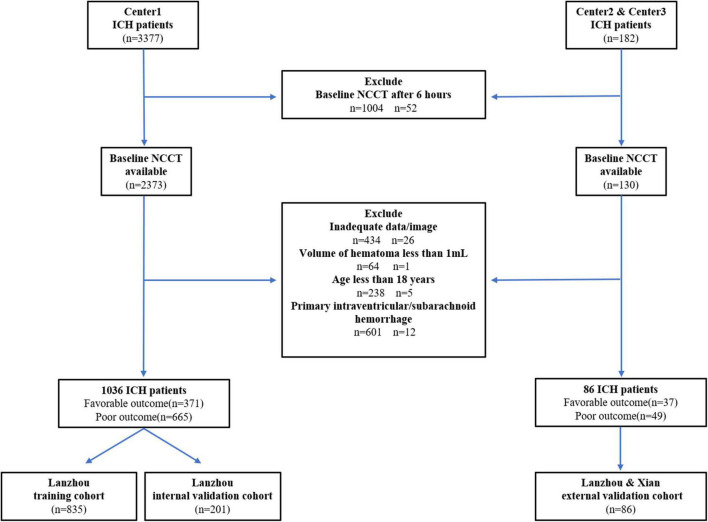
Flowchart of patients’ selection.

We screened consecutive adult patients (aged > 18 years) with spontaneous ICH who underwent baseline CT within 6 h after ICH symptom onset. We excluded patients who underwent surgery before the CT scan, and clinical or laboratory data are incomplete. Additionally, we excluded patients with secondary ICH (brain tumor, trauma, cerebral aneurysm, hemorrhagic transformation of brain infarction, arteriovenous malformation, arteriovenous fistula, and venous malformation). Finally, we included 1,122 patients with ICH [1,036, 25, and 61 patients from center 1 (Lanzhou, China), center 2 (Lanzhou, China), and center 3 (Xian), respectively] from January 2016 to October 2020 (center 1) and from June 2021 to November 2021 (center 2, 3) (Figure 1). Patients from center 1 were divided into the training (2016–2019, *n* = 835) and internal validation cohorts (2020, *n* = 201). Patients from centers 2 and 3 were included in the external validation cohort.

### Clinical and Radiological Characteristics

We collected demographic characteristics, laboratory examinations, and baseline NCCT findings from the Hospital Information System and Picture Archiving and Communication System records of each center. Table 1 shows the laboratory examinations, which were recorded closest to the time of ICH onset. Poor and good 90-day functional outcomes were defined as modified Rankin scale (mRS) scores of 4–6 and 0–3, respectively, which were obtained through standardized telephone interviews or medical records (Qureshi et al., 2016; Hanley et al., 2017; Sprigg et al., 2018). For patients who were lost to follow-up, the final follow-up was considered as the condition on the day of hospital discharge (Ferro et al., 2004). Using a software package (ITK-SNAP version 3.8.0), the volume of interest (VOI) of whole hemorrhages and PHE was manually segmented on axial images (Supplementary Figure 1) by four raters [SL and JD (3 years of clinical experience in neuroradiology); XH and HZ (5 years of clinical experience in neuroradiology)]. ICH locations were classified as lobar (frontal, temporal, parietal, occipital) or deep (hemorrhage originating from the lenticular or caudate nucleus, thalamus, internal or external capsule, and brainstem) (Pasi et al., 2021). Furthermore, each NCCT scan was classified as positive or negative based on the presence of radiological markers (the presence of any heterogeneous type was considered positive for “hypodensity”) (Barras et al., 2009; Boulouis et al., 2016). Midline shift was defined as the maximum midline shift among pineal gland, septum pellucidum, and cerebral falx more than 4 mm on axial images (Yang et al., 2018). The final VOI and radiological markers were adjusted and defined by DW (6 years of diagnostic experience in neuroradiology in an academic full-service hospital). All the aforementioned examiners were blinded to all clinical data and functional outcomes.

**TABLE 1:** Patients’ characteristics in the training or validation cohort.

Characteristic	Lanzhou training cohort *n* = 835	Lanzhou internal validation cohort *n* = 201	External validation cohort *n* = 86	*P*-value
Poor outcome, *n* (%)	533 (63.8)	132 (65.7)	49 (57.0)	0.364
Time, h, median [IQR]	3.0 [2.0, 4.0]*	3.0 [1.0, 4.0]	2.5 [1.0, 4.0]	0.004
**Gender**				
Male, *n* (%)	482 (57.7)	137 (68.2)	52 (60.5)	0.25
Female, *n* (%)	353 (42.3)	64 (31.8)	34 (39.5)	
Age, y, mean ± SD	60 ± 12	60 ± 12	60 ± 15	0.924
**Location**				
Deep, *n* (%)	748 (89.6)	181 (90.0)	72 (82.6)	0.004
Lobar, *n* (%)	87 (10.4)	20 (10.0)	14 (16.3)	
Midline shift, *n* (%)	211 (25.3)	46 (22.8)	12 (14.0)	0.065
IVH, *n* (%)	412 (49.3)	99 (49.3)	40 (46.5)	0.962
SAH, *n* (%)	154 (18.5)	43 (21.4)	16 (18.6)	0.634
Hypodensities, *n* (%)	539 (64.6)	148 (73.6)	49 (57.0)	0.011
ICH volume, ml, median [IQR]	29.2 [12.2, 67.4]	33.1 [16.4, 76.7]	43.8[14.7, 67.1]	0.152
PHE volume, ml, median [IQR]	8.8 [3.8, 19.9]*^#^	16.5 [7.5, 31.4]	16.8 [7.2, 31.7]	<0.001
Temperature, °C, median [IQR]	36.6 [36.5, 36.9]*^#^	36.5 [36.3, 36.7]	36.5 [36.2, 36.6]	<0.001
Smoking, *n* (%)	137 (16.4)	31 (15.4)	25 (29.1)	0.047
SBP, mmHg, median [IQR]	172 [151, 190]	174 [155, 195]	175 [154, 192]	0.556
GCS, mean ± *SD*	10 ± 4	10 ± 4	9 ± 3	0.23
GLU, mmol/L, median [IQR]	7.9 [6.4, 10.0]	7.7 [6.2, 9.8]	8.1 [6.1, 9.1]	0.468
TG, mmol/L, median [IQR]	1.28 [0.82, 2.14]*	1.06 [0.65, 1.74]^+^	1.46 [1.07, 1.84]	0.585
WBC, 10^9^/L, median [IQR]	8.55 [6.28, 11.60]^#^	8.16 [6.39, 11.51]^+^	10.27 [6.89, 13.87]	0.007
NE, 10^9^/L, mean ± *SD*	7.5 ± 4.0^+^	7.3 ± 4.0^#^	9.2 ± 5.4	0.014
LY, 10^9^/L, median [IQR]	1.02 [0.69, 1.59]	1.12 [0.69, 1.76]	1.11 [0.82, 1.59]	0.205
NLR, median [IQR]	6.22 [3.35, 12.27]	5.88 [2.73, 11.62]	7.68 [3.99, 13.28]	0.113
HGB, g/L, median [IQR]	148 [137, 161]^+^	151 [138, 164]^#^	141 [129, 154]	<0.001
INR, median [IQR]	0.98 [0.93, 1.04]*	1.02 [0.97, 1.06]^+^	1.03 [0.95, 1.06]	<0.001

*Data are noted as mean and standard deviation, median and interquartile ranges, or numbers and percentages in parenthesis.*

*Lanzhou training cohort vs. Lanzhou internal validation cohort. ^#^Lanzhou training cohort vs. External validation cohort. ^+^Lanzhou internal validation cohort vs. External validation cohort.

*Time: time from symptom onset to baseline CT, IVH intraventricular hemorrhage; SAH, subarachnoid hemorrhage; ICH, intracerebral hemorrhage; PHE, perihematomal edema; SBP, systolic blood pressure; GCS, Glasgow Coma Scale; GLU, glucose; TG, triglycerides; WBC, white blood cell; NE, neutrophil; LY, lymphocyte; NLR, neutrophil-lymphocyte ratio; HGB, hemoglobin; INR, international normalized ratio.*

### Model Building and Validation

First, we performed univariate analysis to identify potential risk factors, including demographic characteristics, laboratory indicators, and radiological markers, for poor outcomes in patients with ICH. And the variance inflation factor (VIF), a measure for collinearity, was calculated for all features to remove redundant features. Significant variables (*p* < 0.05) and be considered clinically relevant (hemorrhage location) (Roh et al., 2020) were included in multivariate logistic regression analysis to identify independent risk factors for poor outcomes. Subsequently, we developed a model using the independent risk factors as well as GCS and hematoma volume. Finally, we used the internal and external validation cohorts to independently verify the model. We generated the receiver operating characteristic (ROC) curve of the model to evaluate its performance; further, we calculated the area under the ROC curve (AUC). Using the training cohort, we plotted the nomogram for visualizing the developed model. To assess the calibration ability of the model, we plotted a calibration curve for comparing the consistency between the observed and model-predicted functional outcomes. Finally, we performed decision curve analysis (DCA) to evaluate the clinical utility of the model.

### Statistical Analysis

All statistical analyses were performed using R (version 3.2.1), SPSS (version 25.0), and GraphPad Prism (version 9.0.0). Continuous variables are expressed as mean (standard deviation) or median (interquartile range) values while discrete variables are expressed as counts (percentages). One-way analysis of variance, the Kruskal-Wallis test, chi-squared test, two-sample *t*-test, or the Mann-Whitney U test were used for univariate analysis, as appropriate. Statistical significance was set at *P* < 0.05.

## Results

### Patient Characteristics

Table 1 shows the baseline characteristics, which were comparable between the training and validation cohorts. Table 2 shows the risk factors for poor 90-day outcomes. Among the patients with ICH in the three centers, 714 [63.6% (training, 533 (63.8%); internal validation, 132 (65.7%); external validation 49 (57.0%)] had poor 90-day outcomes, with no significant among-cohort differences. There was a significant difference in the time from symptom onset to admission between the training cohort [3.0 (2.0, 4.0)] and the internal validation cohort [3.0 (1.0, 4.0)]. However, in the training cohort, there was no significant difference between the poor and good outcome groups. There were no significant among-cohort differences in age; moreover, the poor outcome group was older than the good outcome group (63 ± 12 vs. 56 ± 11 years, *p* < 0.001). The validation cohort had a larger PHE volume than the training cohort. Moreover, the poor outcome group had a larger PHE volume than the good outcome group [13.3(5.7, 25.5) vs. 5.2 (2.3, 11.6) ml, *p* < 0.001]. There were no significant among-cohort differences in the ICH volume and Glasgow Coma Scale (GCS) scores; however, they showed significant between-group differences (*p* < 0.001). Further, there were significant between-group differences in laboratory indicators such as glucose (GLU), white blood cell (WBC), neutrophil (NE), and neutrophil-lymphocyte ratio (NLR) (Supplementary Figure 2).

**TABLE 2:** Univariate analysis for poor outcome in the training cohort.

Characteristic	Lanzhou training cohort		
	Poor outcome	Good outcome	*P*-value
Poor outcome, *n* (%)	533 (63.8)	302 (36.2)	
Time, h, median [IQR]	3.0 [2.0, 4.0]	3.0 [1.5, 4.0]	0.915
**Gender**			
Male, *n* (%)	311 (64.5)	171 (35.5)	0.662
Female, *n* (%)	222 (62.8)	131 (37.2)	
Age, y, mean ± *SD*	63 ± 12	56 ± 11	<0.001
**Location**			
Deep, *n* (%)	478 (89.6)	270 (89.4)	0.907
Lobar, *n* (%)	55 (10.4)	32 (10.6)	
Midline shift, *n* (%)	193 (91.5)	18 (8.5)	<0.001
IVH, *n* (%)	342 (83.0)	70 (17.0)	<0.001
SAH, *n* (%)	136 (88.3)	18 (11.7)	<0.001
Hypodensities, *n* (%)	376 (69.6)	163 (30.4)	<0.001
ICH volume, ml, median [IQR]	51.6 [20.5, 88.8]	14.4 [5.6, 25.8]	<0.001
PHE volume, ml, median [IQR]	13.3 [5.7, 25.5]	5.2 [2.3, 11.6]	<0.001
Temperature, °C, median [IQR]	36.7 [36.5, 36.9]	36.6 [36.4, 36.8]	0.014
Smoking, *n* (%)	93 (67.8)	44 (32.2)	0.415
SBP, mmHg, median [IQR]	178 [156, 193]	165 [148, 185]	<0.001
GCS, mean ± SD	8 ± 4	13 ± 2	<0.001
GLU, mmol/L, median [IQR]	8.5 [6.8, 10.8]	6.8 [5.9, 8.7]	<0.001
TG, mmol/L, median [IQR]	1.3 [0.8, 2.1]	1.2 [0.7, 2.0]	0.349
WBC, 10^9/L, median [IQR]	8.90 [6.65, 12.75]	7.76 [5.70, 10.26]	<0.001
NE, 10^9/L, mean ± *SD*	8.1 ± 4.3	6.5 ± 3.1	<0.001
LY, 10^9/L, median [IQR]	1.02 [0.65, 1.64]	1.02 [0.77, 1.51]	0.491
NLR, median [IQR]	7.47 [3.47, 13.68]	5.29 [3.28, 9.97]	<0.001
HGB, g/L, median [IQR]	148 [137, 162]	148 [137, 159]	0.435
INR, median [IQR]	0.98 [0.93, 1.05]	0.98 [0.93, 1.03]	0.668

*Data are noted as mean and standard deviation, median and interquartile ranges, or numbers and percentages in parenthesis.*

*Time: time from symptom onset to baseline CT, IVH intraventricular hemorrhage, SAH, subarachnoid hemorrhage; ICH, intracerebral hemorrhage; PHE, perihematomal edema; SBP, systolic blood pressure; GCS, Glasgow Coma Scale; GLU, glucose; TG, triglycerides; WBC, white blood cell; NE, neutrophil; LY, lymphocyte; NLR, neutrophil-lymphocyte ratio; HGB, hemoglobin; INR, international normalized ratio.*

### Computed Tomography

Hematoma location did not significantly affect patient outcome. There was no difference in the proportion of deep-located hematoma between the poor and good outcome groups [478 (63.9%) vs. 270 (89.4%), *p* = 0.907]. There were no among-cohort differences in the midline shift, IVH, and SAH; however, their appearance usually led to poor outcomes. The proportion of hypodensities showed significant among-cohort and between-group differences.

### Predictors of Poor Outcome

Univariate analysis showed that age, midline shift, IVH, SAH, hypodensities, ICH volume, PHE volume, temperature, systolic blood pressure (SBP), GCS score, GLU, WBC, NE, and NLR were associated with poor 90-day prognosis (*p* < 0.05). After the VIF filtering and multivariate logistic regression analysis revealed the following independent risk factors (*p* < 0.05): age (OR = 1.89; 95% CI, 1.55–2.33; *P* < 0.001), ICH volume (OR = 6.99; 95% CI,4.44–11.43; *P* < 0.001), GCS (OR = 0.35; 95% CI, 0.27–0.44; *P* < 0.001), and deep hematoma location (OR = 2.34; 95% CI, 1.23–4.50; *P* = 0.010) (Table 3 and Supplementary Figure 3).

**TABLE 3:** Multivariate analysis for poor outcome in the training cohort.

Variables	OR	95%CI	*P*-value
Age, y, mean ± SD	1.89	1.55–2.33	<0.001
Deep	2.34	1.23–4.50	0.010
ICH volume, ml, median [IQR]	6.99	4.44–11.43	<0.001
GCS, mean ± SD	0.35	0.27–0.44	<0.001

*ICH, intracerebral hemorrhage; PHE perihematomal, edema; GCS, Glasgow Coma Scale; WBC, white blood cell; NE, neutrophil; NLR, neutrophil-lymphocyte ratio; OR, odds ratio; CI, confidence interval.*

**FIGURE 2:**
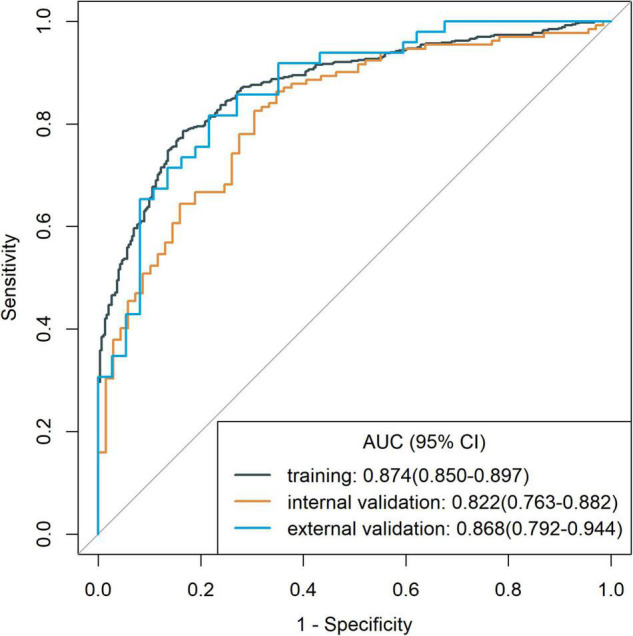
Receiver operating characteristic curves of the model for assessing 90-day clinical functional outcome in the training cohort and validation cohorts.

**FIGURE 3:**
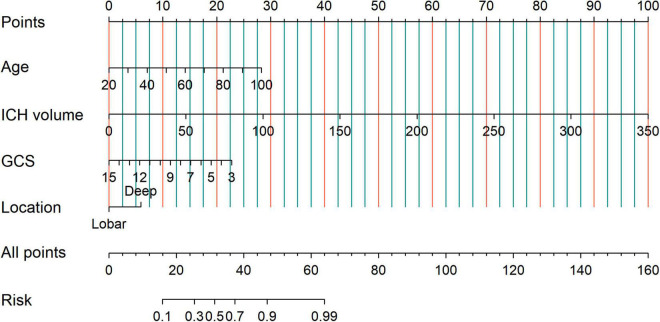
The clinical nomogram for assessing 90-day clinical functional outcome.

**FIGURE 4:**
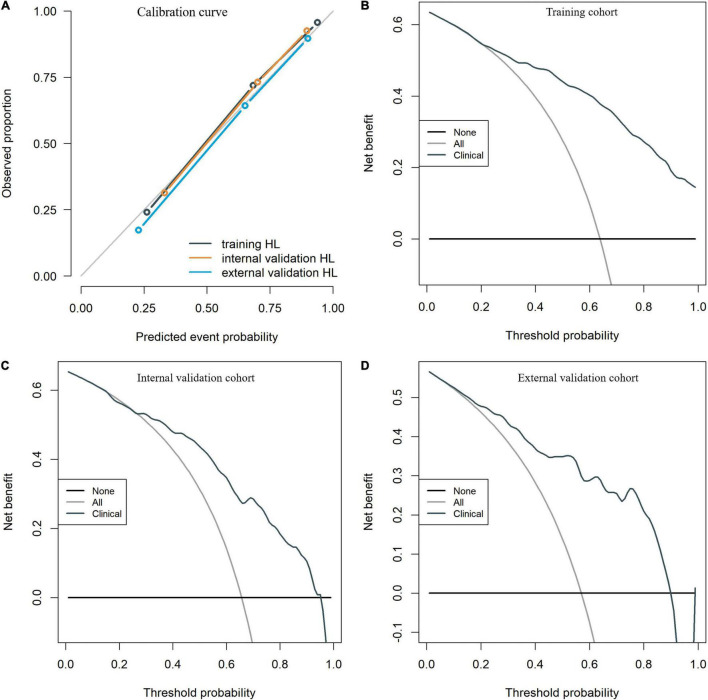
The calibration curves for the clinical nomogram in the training and validation cohorts **(A)**. The decision curve for the clinical nomogram in the training **(B)** and validation cohorts **(C,D)**.

To identify patients with poor prognosis, the model showed an AUC of 0.874 (specificity, 0.834; sensitivity, 0.786), 0.822 (specificity, 0.696; sensitivity, 0.759), and 0.868 (specificity, 0.649; sensitivity, 0.918) in the training, internal validation, and external validation cohorts, respectively (Figure 2). We developed a nomogram for visualizing the model results (Figure 3). The calibration curve for the probability of poor 90-day outcomes showed a favorable predictive performance that was satisfactorily consistent with the ideal curve (Figure 4A). DCA revealed that the model had good clinical utility at predicting poor 90-day outcomes in patients with ICH in all cohorts (Figures 4B–D).

## Discussion

Compared with traditional single clinical indicators to evaluate the prognosis, nomogram allow the development of potential biologically and clinically integrated models and fulfill our drive toward personalized medicine (Balachandran et al., 2015). Rapid computation through user-friendly digital interfaces, aid clinical decision making in patients with ICH, such as intensive blood pressure reduction or resumption of anticoagulants therapy (Kuramatsu et al., 2015; Li et al., 2020). We established and independently validated a nomogram model for predicting poor 90-day outcomes. The nomogram model showed favorable performance in predicting poor 90-day outcomes in all cohorts with a large AUC value. This model can be solely completed based on findings obtained on admission and can be evaluated in the hyperacute ICH phase. Additionally, it is easy to use without the need for extensive experience in recognizing imaging signs, which is suitable for inexperienced first-line clinicians. Furthermore, all variables for the constitutive model were objective and could be easily obtained in the routine examinations upon admission. Taken together, our model was simple, efficient, and objective. There remains no drug for treating ICH that is approved by the Food and Drug Administration; moreover, no randomized controlled trials have shown a neurosurgical intervention that can improve the curative effect compared with conservative treatment (O’Carroll et al., 2021). There is a need for accurate prediction of outcomes in patients with acute ICH, which could help physicians promptly implement effective interventions as well as improve patients’ awareness regarding active rehabilitation training (Hemorrhagic Stroke Academia Industry (HEADS) Roundtable Participants, 2018).

In the current study, several predictive factors for poor 90-day prognosis were identified, including the GCS score, persistent hyperglycemia, blood pressure, and age (Rådholm et al., 2015; Wu et al., 2017; Chung et al., 2018; Shoamanesh et al., 2018; Lo and Teitelbaum, 2020). Further, we confirmed that age and GCS score were independent predictors of poor 90-day outcomes in patients with ICH. Specifically, an age > 63 years increased the risk of a poor prognosis since older patients often present related diseases, including high blood pressure and heart disease, that affect outcomes in patients with ICH. The “GSC” score is an important evaluation index for neuron injury (Teasdale et al., 2014) and is widely used to assess “overall” brain damage. It assesses three aspects regarding the patient’s responsiveness (eye, verbal, and motor responses). In the poor outcome group, the mean GCS score was 8, which suggests that severe disturbance of consciousness and serious nerve injury lead to an unfavorable prognosis. Consistent with our findings, a previous study showed that a low GCS score on admission could predict poor outcomes in patients with ICH (Zhang et al., 2015). Although temperature and SBP showed significant between-group differences, they were not independent predictors of prognosis. This could be attributed to the fact that they are examined during the rapid transport of patients to the hospital, which could lead to some inaccuracies. Inflammatory reactions are a significant element of the ICH pathological process and may promote secondary brain injury through various complex pathological mechanisms, which may lead to an unfavorable prognosis. The NLR, which comprehensively reflects two different but complementary immune pathways, is an independent predictive factor for adverse outcomes in patients with cardiovascular disease (Angkananard et al., 2018). We found that WBC, NE, and NLR, which can be easily obtained, were differences between different outcome groups. However, after the VIF filtering and multivariate logistic regression analysis, these are not an independent risk factor for poor outcome. This result may be due to the inflammatory reactions are dynamic, which participated in the whole process of tissue damage and repair. Then, the measured value of a single point in time is lacking stability on the prognosis. Hyperglycemia may cause secondary neuron damage by exacerbating the oxidative damage in patients with cerebral inflammatory disease (Stead et al., 2010). However, our findings did not indicate that blood glucose can predict prognosis, which could be attributed to our application of a single glucose measurement that did not account for potential glucose fluctuations after ICH. While inflammation is important in repairing tissue after insult, it often results in an exacerbation of tissue injury. As our results show, abnormal increases in WBC, NE, NLR, and GLU are associated with poor prognosis. This may be the reason for the therapeutic strategies that target the immune system after ICH (Shao et al., 2019). The hematoma volume is an important index of poor prognosis. Hematoma brain injury is mainly caused by shear injury and the space-occupying effect (Mittal and LacKamp, 2016; Fang et al., 2019). The space-occupying effect, inflammatory reactions, coagulation cascade, prothrombin activation, and lysis of red blood cells lead to PHE. The mechanisms underlying PHE formation differ across the various stages. Changes in PHE (<6 h) are mainly mediated by blood clot retraction and serum protein accumulation in surrounding parenchymatous tissues (Ironside et al., 2019; Chen et al., 2021). We observed between-group differences in the PHE volume; however, it showed no statistical significance in the logistic regression model. The primary reason underlying this finding is unclear; additionally, there have been inconsistent findings regarding the prognostic effects of PHE (Sprügel et al., 2019; Selim and Norton, 2020). The relationship between PHE and clinical outcomes is complicated by numerous confounders, including the ICH volume, PHE quantification method, and timing of PHE measurement (Ironside et al., 2019). However, aggravation of the space-occupying effect resulting from the PHE volume is an important objective factor in neuronal injury. CT is the gold standard diagnostic method for ICH (Latchaw et al., 2009). Several CT-based imaging markers using direct or indirect methods (e.g., prediction of hematoma expansion) have been proposed for assessing outcomes in patients with ICH (Li et al., 2017, 2018; Law et al., 2020; Hostettler et al., 2021; Singh et al., 2021). CT imaging biomarkers based on the hematoma density and shape are largely subjective due to vague descriptions and definitions, which subsequently reduces inter-observer agreement during visual assessment (Ma et al., 2019). Therefore, to ensure maximal interrater reliability and reproducibility, we included only one CT imaging maker, which was derived by simply dichotomizing NCCT scans according to the presence or absence of hypodensity. Nonetheless, we found that the presence of hypodensity was not an independent risk factor for poor outcomes, which indirectly reflects the complex pathological mechanism underlying hypodensities. Detailed classification of hypodensity signs may improve accuracy; however, this had limited clinical applicability among doctors with insufficient clinical experience. Several recent studies (Nawabi et al., 2021; Pszczolkowski et al., 2021; Song et al., 2021) have shown that radiomics, which applies machine learning (ML) algorithm, may be a more efficient and objective method for evaluating outcomes in patients with ICH. The ML method for evaluating the prognosis of patients with ICH remains in its infancy and has unstable models. Moreover, most previous studies were small-scale single-center studies; therefore, there is a need for large-scale multi-center studies to validate this method. Nonetheless, the ML method remains a promising research tool for evaluating ICH that could reveal more high-dimensional features that are not visible to the naked eye on images (Gillies et al., 2016).

### Limitations

We acknowledge that our study has several important shortcomings. First, since the time from symptom onset to baseline CT in most patients with ICH was <6 h, our study design did not include patients whose duration was ≥6 h. This limits the application scope of our model. Second, since this was an observational study, the clinical care could have varied across patients, including early withdrawal of care and lackluster rehabilitation among patients limited by their educational level and economic situation, and there was an inevitable risk of recall bias. Therefore, in this study, we used data sets from Center 1 in 2020 years, and data sets from Center 2 and 3 (more recent date- June 2021 to November 2021) as internal validation and external validation, respectively, to verify the influence of these factors on the model. The results showed that the prediction performance of our model was stable. Third, since center 1 is a local stroke center, and center 2 and 3 have a small number of cases due to their medical system reasons, the patients’ enrollment is imbalance. Fourth, to ensure interrater reliability and reproducibility, we did not include specific imaging signs or quantitative indicators (e.g., island, black hole signs and grading, and classification of hydrocephalus), which require evaluation by physicians experienced in neuroimaging. Additionally, we did not use ML. Future studies on radiomics and/or deep learning (DL), which may have better performance than our clinical model, are warranted.

## Conclusion

Using laboratory indexes and clinical factors, we developed and independently validated a clinical model for predicting a poor 90-day prognosis within 6 h after ICH onset. Our model showed favorable predictive performance; moreover, it is objective and easy to use. Our findings could facilitate the identification of patients at risk of poor outcomes as well as inform future research on effective treatments and prognosis improvement.

## Data Availability Statement

All data generated or analyzed during this study are included in this published article.

## Ethics Statement

The studies involving human participants were reviewed and approved by the Institutional review boards (IRBs) Lanzhou University Second Hospital. Written informed consent for participation was not required for this study in accordance with the national legislation and the institutional requirements.

## Author Contributions

XYH, DW, QYZ, YQM, and JLZ designed the method, contributed to the acquisition of data, and prepared the manuscript. XYH, DW, SLL, and JLR designed the method, aided in data analysis, and revised and approved the manuscript. SLL, HZ, JD, JJY, MX, HZX, FKL, HYZ, YJX, LY, YCH, MYY, and QZ aided in data acquisition and interpretation. All authors contributed to the article and approved the submitted version.

## Conflict of Interest

JLR was employed by the GE Healthcare. The remaining authors declare that the research was conducted in the absence of any commercial or financial relationships that could be construed as a potential conflict of interest.

## Publisher’s Note

All claims expressed in this article are solely those of the authors and do not necessarily represent those of their affiliated organizations, or those of the publisher, the editors and the reviewers. Any product that may be evaluated in this article, or claim that may be made by its manufacturer, is not guaranteed or endorsed by the publisher.
