# Supplementary material for: Development and Validation of a Clinical-Based Signature to Predict the 90-Day Functional Outcome for Spontaneous Intracerebral Hemorrhage
Source: Front Aging Neurosci. 2022 May 9;14:904085. doi: 10.3389/fnagi.2022.904085 (PMC9125153; doi:10.3389/fnagi.2022.904085)
Supplement: Supplementary file 1 [file Presentation_1.pdf]

Supplementary

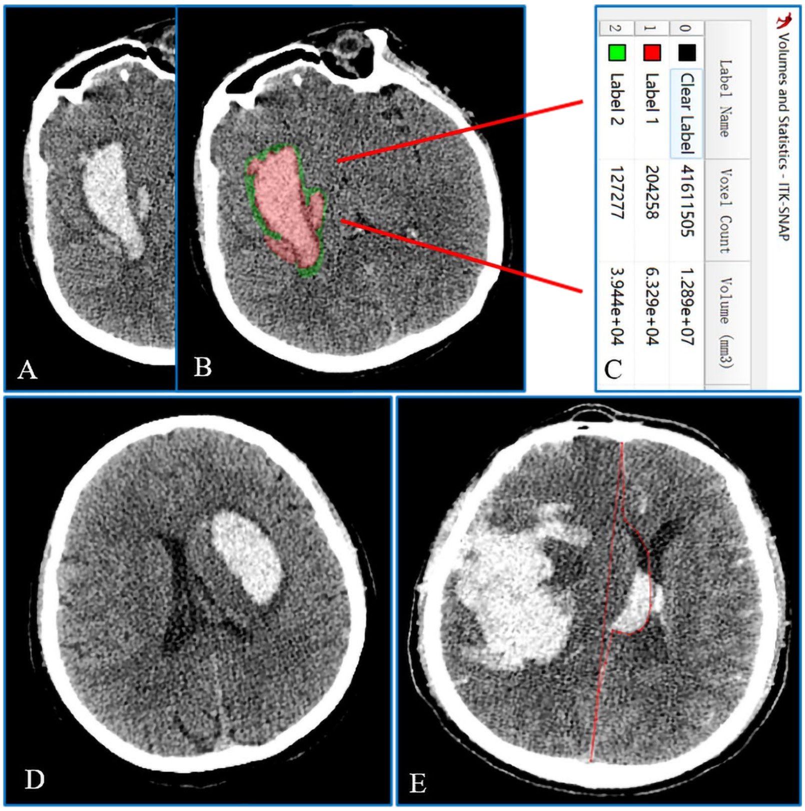

FIGURE 1 | Manually delineate and measure ICH and PHE volumes（A-C）

(D) example of homogeneous and regular ICH (be considered negative for hypodensity) (E) example of positive for hypodensity (the presence of any o types of hypodensities), midline shift (red line), intraventricular hemorrhage (IVH), and subarachnoid hemorrhage (SAH).

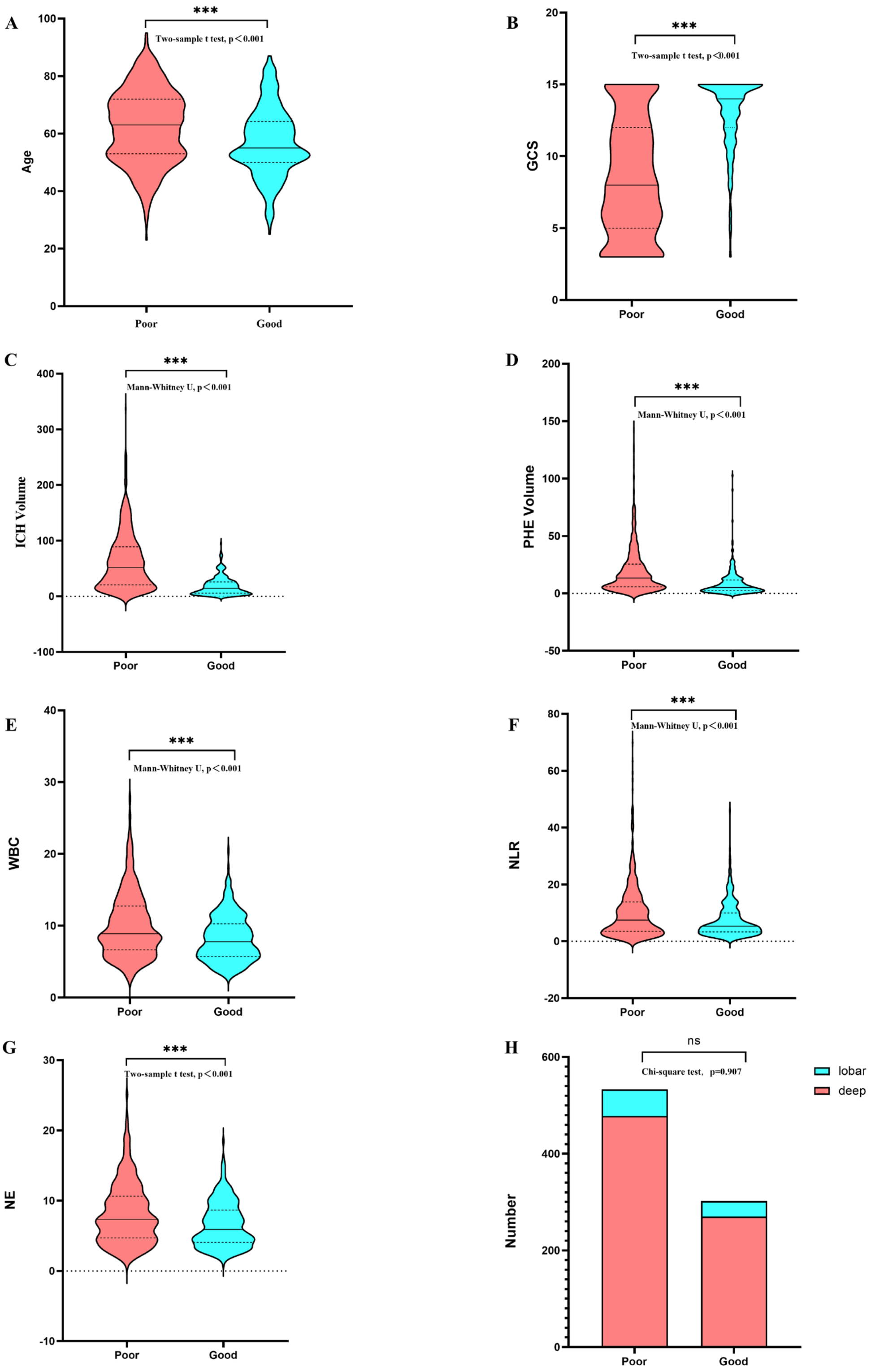

FIGURE 2 | The differences between the Poor outcome and good outcome groups. ICH intracerebral hemorrhage, PHE perihematomal edema, GCS Glasgow Coma Scale, WBC white blood cell, NE neutrophil, NLR neutrophil-lymphocyte ratio.

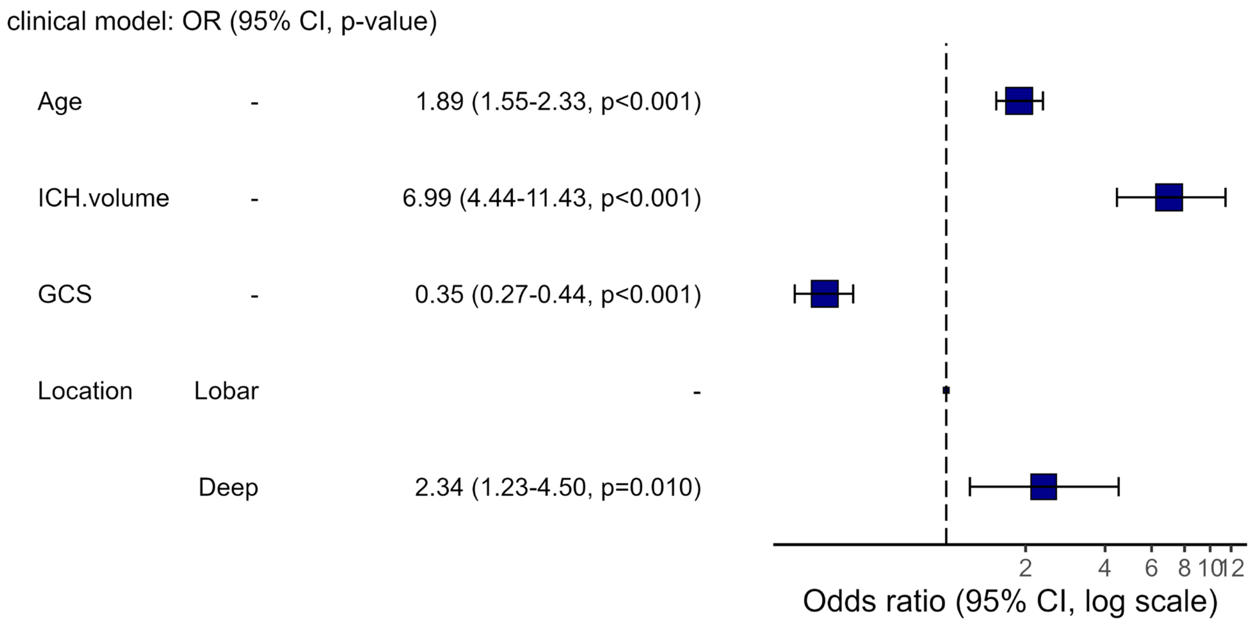

FIGURE 3 | Forest plot of odds ratio (OR) for multivariate analysis for poor outcome in the training cohort. ICH intracerebral hemorrhage, GCS Glasgow Coma Scale,, OR odds ratio, CI confidence interval.
